# Selection signatures for local and regional adaptation in Chinese Mongolian horse breeds reveal candidate genes for hoof health

**DOI:** 10.1186/s12864-023-09116-8

**Published:** 2023-01-19

**Authors:** Haige Han, Imtiaz A. S. Randhawa, David E. MacHugh, Beatrice A. McGivney, Lisa M. Katz, Manglai Dugarjaviin, Emmeline W. Hill

**Affiliations:** 1 Inner Mongolia Key Laboratory of Equine Genetics, Breeding and Reproduction, College of Animal Science, Equine Research Centre, Inner Mongolia Agricultural University, Hohhot, 010018 China; 2 Animal Genetics Laboratory, School of Veterinary Science, University of Queensland, Brisbane, Australia; 3 UCD School of Agriculture and Food Science, University College Dublin, Belfield, Dublin, D04 V1W8 Ireland; 4 UCD Conway Institute of Biomolecular and Biomedical Research, University College Dublin, Belfield, Dublin, D04V1W8 Ireland; 5 Plusvital Ltd, The Highline, Dun Laoghaire Business Park, Dublin, A96 W5T3 Ireland; 6 UCD School of Veterinary Medicine, University College Dublin, Belfield, Dublin, D04V1W8 Ireland

**Keywords:** Chinese Mongolian horse, Selection signatures, Hoof health, Regional adaptation

## Abstract

**Background:**

Thousands of years of natural and artificial selection since the domestication of the horse has shaped the distinctive genomes of Chinese Mongolian horse populations. Consequently, genomic signatures of selection can provide insights into the human-mediated selection history of specific traits and evolutionary adaptation to diverse environments. Here, we used genome-wide SNPs from five distinct Chinese Mongolian horse populations to identify genomic regions under selection for the population-specific traits, gait, black coat colour, and hoof quality. Other global breeds were used to identify regional-specific signatures of selection.

**Results:**

We first identified the most significant selection peak for the Wushen horse in the region on ECA23 harbouring DMRT3, the major gene for gait. We detected selection signatures encompassing several genes in the Baicha Iron Hoof horse that represent good biological candidates for hoof health, including the *CSPG4*, *PEAK1*, *EXPH5*, *WWP2* and *HAS3* genes. In addition, an analysis of regional subgroups (Asian compared to European) identified a single locus on ECA3 containing the *ZFPM1* gene that is a marker of selection for the major domestication event leading to the DOM2 horse clade.

**Conclusions:**

Genomic variation at these loci in the Baicha Iron Hoof may be leveraged in other horse populations to identify animals with superior hoof health or those at risk of hoof-related pathologies. The overlap between the selection signature in Asian horses with the DOM2 selection peak raises questions about the nature of horse domestication events, which may have involved a prehistoric clade other than DOM2 that has not yet been identified.

**Supplementary Information:**

The online version contains supplementary material available at 10.1186/s12864-023-09116-8.

## Introduction

For millennia horses have played a central role in the nomadic life of Mongolic ethnic groups. For Mongols, known as an ‘ethnic group on horseback’, the horse has had considerable cultural and economic significance with the horse relied on for transportation, use in warfare and as a source of food (meat and mare’s milk). Horse racing is recognised as one of the ‘three manly games’ and a wealth of cultural literary accounts in songs and poems relate to horses. Today, the Mongolian horse is recognised as one of the oldest known horse breeds in the world [1]. Present-day Mongolian horses are mainly distributed in parts of northeast and north China (mainly Inner Mongolia), Mongolia and some areas of eastern Russian [2]. For the present study, we focused on the Mongolian horse and derived breeds from the Inner Mongolia Autonomous Region (IMAR), China, hereafter referred to as the Chinese Mongolian horse.

Chinese Mongolian horses are found in the IMAR, western parts of the provinces of Heilongjiang, Jilin and Liaoning and some parts of the Xinjiang Uygur Autonomous Region (XUAR) [1, 3]. The earliest written record of the Chinese Mongolian horse is found in the Book of Han which records that “as early as King Yao’s and Shun’s time (2,377 – 2,178 BC) this horse lived in the river basin depending on the natural pastures along with cattle and sheep” [4]. It has been noted that historical records may have existed before horses were ridden because it is considered likely that the Chinese Mongolian horse was domesticated ~ 4 kya in northern China near the present-day Mongolian border [1]. At present, the Chinese Mongolian horse is generally classified as a breed in toto; however, as a result of long-term selection for adaptation to local environments by herdsmen, four phenotypically distinct sub-types have evolved: Wushen (desert type), Wuzhumuqin (steppe type), Baicha Iron Hoof (mountain type) and Baerhu (steppe type). Other breeds (e.g. Sanhe, Keerqin and Abaga Black) found in Inner Mongolia have been developed from local Mongolian stock following crossbreeding with imported horses from Europe and elsewhere.

Horses in Inner Mongolia have adapted to harsh environments, grazing year-round without supplemental feeding even when the ground is covered with snow and experiencing temperatures below − 40 °C in severe winters. Generally, they display traits for adaptation to extreme cold and dry hot conditions. Several metabolic, morphological, and physiological adaptations have been proposed to have evolved in Chinese Mongolian populations in response to their environment. For instance, the horses exhibit morpho-anatomical adaptations to the cold winter, developing extremely hairy coats in the winter that are shed by late spring before the hot summer. In addition, anecdotally it has been suggested that these horses exhibit reduced susceptibility to certain infectious diseases [3]. Some Chinese Mongolian horse populations have been further shaped by local herdsmen for traits such as alternative gait (Wushen), black coat colour (Abaga Black) and strong hooves (Baicha Iron Hoof). We have recently described the fine-scale characterization of the population genetic structure of five Chinese Mongolian horse populations using genome-wide single-nucleotide polymorphism (SNP) data [5]. In a principal component analysis (PCA), Abaga Black was the most genetically distinct from the other four populations while the Baicha Iron Hoof was partitioned into two clusters, one of which was similar to the Sanhe, Wuzhumuqin and Wushen populations. Although Wuzhumuqin, Wushen and Sanhe clustered in the first two principal components (PCs), the pairwise fixation index (*F*_ST_) value of 0.005 estimated for the most closely related populations, Wushen and Wuzhumuqinm, was higher than the pairwise *F*_ST_ value (0.002) estimated for Paint and Quarter Horse, which are recognised as distinct breeds [6, 7]. Therefore, we hypothesised that long-term selection pressure for population-specific traits (i.e. gaitedness, strong hooves and black coat colour) may have contributed to the observed genomic diversity among the Chinese Mongolian populations. Furthermore, these five Chinese Mongolian as well as two other Asian populations (the Mongolian horse populations in Mongolia and Tuva) were genetically similar to each other but distinct from other global horse populations and were positioned between two large regional groups (European and American/Iberian/Middle Eastern) in the PCA analysis [5]. These results therefore lead to the hypothesis that natural and artificial selection will have left signatures in the Chinese Mongolian populations for long-term environmental adaptation when compared to other global horse populations.

Here, we report novel genome-wide signals of selection in the Baicha Iron Hoof population identified by comparison with four other Chinese Mongolian populations and signals for adaptation to regional environmental conditions in Asian horses compared with European as well as American/Iberian/Middle Eastern horse populations by applying the composite selection signals (CSS) approach [8] that summarises multiple tests for selection using genome-wide SNP data for 30 global horse breeds, including five Chinese Mongolian populations grouped by phenotype.

## Results

### Selection signals for alternative gait in Wushen horse

**Fig. 1:**
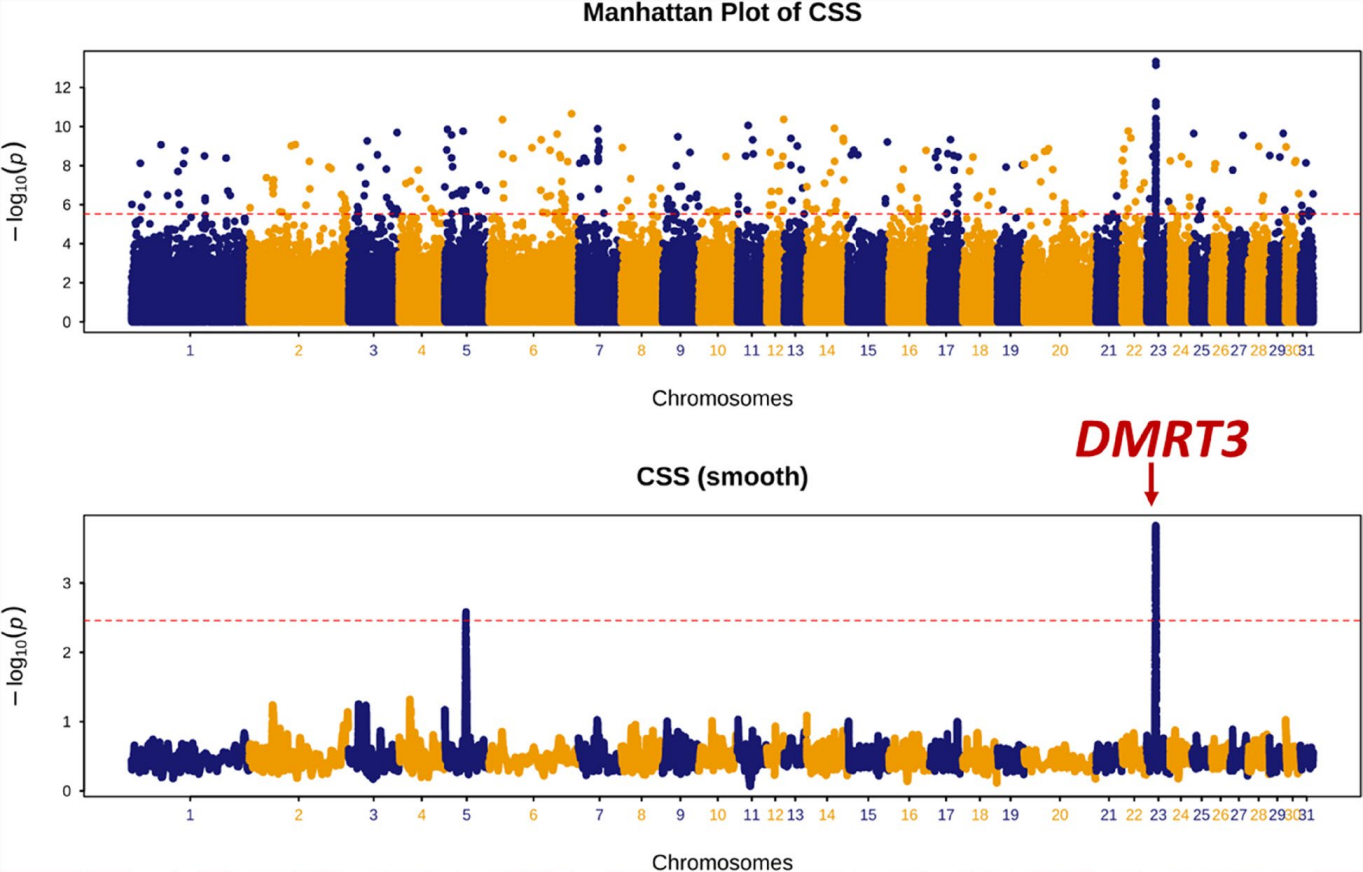
A chromosome-wide plot of composite selection signals (CSS) scores for the Gaited (*n* = 22 Wushen) vs. Non-gaited (*n* = 78 other Mongolian) horses in dataset cohort. 1) Unsmoothed results, 2) Smoothed results, obtained by averaging the CSS scores of SNPs within 100 kb sliding windows. The dashed red lines indicate the genome-wide 0.1% thresholds of the empirical scores. The red arrow indicates the location of *DMRT3* on ECA23

The ability to pace (a two-beat lateral gait) has been selected in Wushen horses by local herdsmen since pacing improves the comfort of riding, particularly over long distances. In addition, Wushen horses are bred to race with a paced gait since gaited horse racing is a popular type of racing in Inner Mongolia. The CSS test approach successfully identified the *DMRT3* gene on ECA23, that has a well-established major effect on alternative gait (pace) [9] by comparing Wushen (*n* = 22) to the four other Chinese Mongolian populations (*n* = 78) (Fig. 1, Table S10).

### Selection signals for black coat colour in Abaga Black horse

Contrary to the results for gaitedness, the genomic region containing the *ASIP* gene on ECA22 was not immediately evident as a selection signature in Abaga Black, when Abaga Black (*n* = 15) horses were compared with other Mongolian horses (*n* = 85) (Fig. S1, Table S2 and S10). The CSS component statistics for SNPs within or proximal to the *ASIP* locus were therefore examined in more detail, but none of the three statistics (ΔSAF, *F*_ST_ and XP-EHH) was significant for any proximal SNPs using the threshold for significance among the top 0.1% SNPs (data not shown). However, by relaxing the threshold to include the top 1% SNPs, there was evidence for variation at the ECA22 locus containing *ASIP* (Fig. S2, Table S3).

### Selection signals for ‘Iron Hoof’

**Fig. 2:**
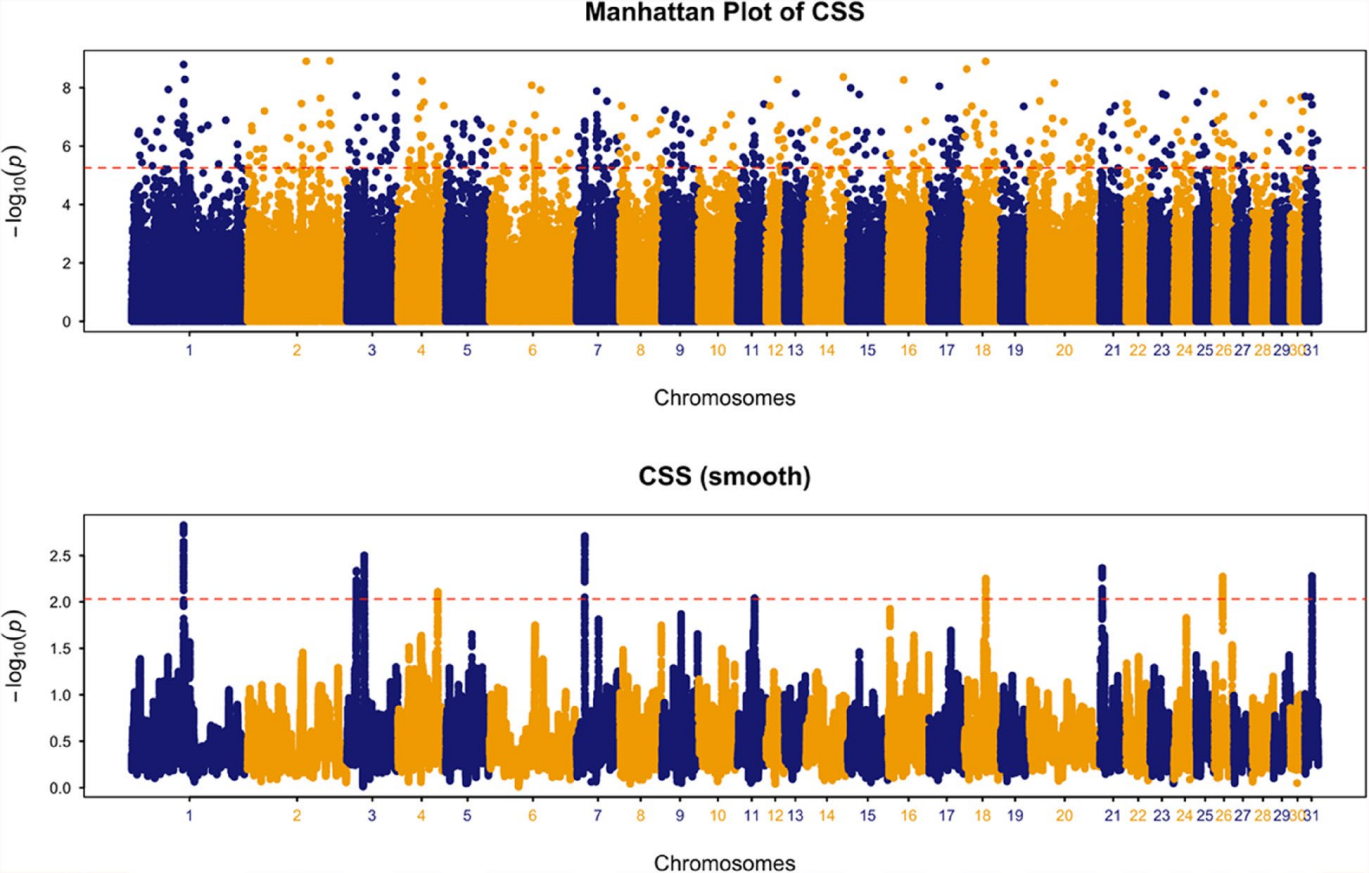
A chromosome-wide plot of the composite selection signals (CSS) scores for Iron Hoof (*n* = 19) vs. Non-Iron Hoof (*n* = 81) in the dataset cohort. 1) Unsmoothed results, 2) Smoothed results, obtained by averaging the CSS scores of SNPs within 100 kb sliding windows. The dashed red lines indicate the genome-wide 0.1% thresholds of the empirical scores. Ten genomic regions with clusters of significant SNPs among the top 0.1% SNPs were identified on ECA1, ECA3, ECA4, ECA7, ECA11, ECA18, ECA21, ECA26 and ECA31

The Baicha Iron Hoof horse is the mountain-type of Chinese Mongolian horse and it is also commonly known simply as the ‘Iron Hoof’ for its strong, tough hooves. To test the hypothesis that the ‘Iron Hoof’ trait is underpinned by genes on which positive selection is acting, we performed CSS for the comparison of the Baicha Iron Hoof (*n* = 19) versus other Chinese Mongolian horses (*n* = 81). Genome-wide distribution of the smoothed CSS (−log_10_*P*) for the comparison identified 10 genomic regions with clusters of significant SNPs among the top 0.1% SNPs on ECA1, ECA3, ECA4, ECA7, ECA11, ECA18, ECA21, ECA26 and ECA31(Fig. 2, Table S4). In the selection peaks and their flanking regions, a total of 193 genes were identified (Table S4). Genes within selection peaks and flanking regions with functions that may be associated with hoof health are summarized in Table 1. The highest ranked region by CSS score (including the flanking region) spanned ~ 1.44 Mb on ECA1 and contained the genes *CSPG4*, *PEAK1*, *SEMA7A*, *CSK* and *PSTPIP1* (also known as *CD2BP1*). The second ranked region on ECA7 flanked the *EXPH5* gene. Furthermore, six candidate genes were identified on ECA3; *WWP2*, *PSMD7*, *NQO1*, *NOB1*, *NFAT5* and *HAS3*. Additionally, we identified several genes (*SEMA7A* [10], *CSK* [11], *GUCY1A2* [12], *EMCN* [13], *OPRM1* [14]) that are associated with rheumatoid arthritis (RA).

**Table 1:** Genes within selection peaks and flanking regions with functions that may be associated with hoof health were detected in the Baicha Iron Hoof horse

ECA	Region (Mb)	Top 0.1% SNPs (*n*)	CSS value	Cluster rank	Candidate genes	Gene function
1	119.28–119.72	88	2.83	1	*CSPG4*, *PEAK1*	Matrix metalloproteinase 2 activity
					*SEMA7A, CSK, PSTPIP1*	Rheumatoid arthritis
7	16.77–17.08	56	2.71	2	*GUCY1A2*	Rheumatoid arthritis
					*EXPH5*	Inherited Skin Fragility
3	39.62–40.1	65	2.50	3	*EMCN*	Rheumatoid arthritis
3	20.14–21.01	104	2.34	5	*WWP2*	Plantar fibromatosis
					*TERF2*	Dyskeratosis congenita
					*PSMD7*	Ankylosing spondylitis
					*NQO1*	Injury and inflammation
					*NOB1*	Osteosarcoma
					*NFAT5*	Inflammatory arthritis
					*HAS3*	Hyaluronan metabolism in human keratinocytes and atopic dermatitis skin
31	12.94–13.16	53	2.28	6	*OPRM1*	Rheumatoid Arthritis

### Selection signals relating to geographic origin / adaptation

To understand genomic variation contributing to regional adaptation we searched for selection signals in the Asian populations (*n* = 133) that distinguish them from European (*n* = 191) and America/Middle Eastern breeds (*n* = 281). To assess functional significance of genes in selected regions, we assigned functional annotation to all genes in the regions defined by the top 0.1% SNPs (including those with < 5 SNPs) using the DAVID functional annotation tool [15] as well as the GeneCards [16].

The genome-wide distribution of the smoothed CSS (−log_10_*P*) for Asian versus American/Middle Eastern population groups revealed six genomic regions on ECA6, ECA17, ECA21, ECA26 and ECA27 exhibiting signatures of selection (Table S5, Fig. S3). These six regions with flanking regions contained 113 genes including those associated with deafness, immune response, response to cold, intellectual disability, nervous system development, mitochondria, muscular dystrophy, muscle contraction, heart development, spermatogenesis, heart contraction, and brain development (Table 2, Table S6).

**Table 2:** Genes with functional significance identified in the selection peaks and flanking regions in the Asian versus American/Middle Eastern horse cohorts

ECA	Region (Mb)	CSS value	Candidate genes	Function
6	82.81–82.81	2.16	*MSRB3*, *LLPH*, *HELB*	Deafness
			*IRAK3*, *HMGA2*, *CAND1*	Immune response
17	57.06–57.22	1.64	*SLITRK6*	Deafness
21	2.62–3.62	1.72	*IL12RB1*, *BST2*, *JAK3*, *IFI30*, *UBA52*, *AP1M1*	Immune response
			*SLC27A1*	Response to cold
			*KCNN1*, *SIN3B*	Intellectual Disability
			*TMEM59L*, *NR2F6*, *MAP1S*, *NCAN*, *HAPLN4*, *CHERP*	Nervous system development
			*YJEFN3*, *MRPL34*, *MPV17L2*, *GTPBP3*, *SLC25A42*, *NWD1*, *NDUFA13*, *HSH2D*, *FAM32A*	Mitochondria
			*TMEM38A*	Muscular Dystrophy
			*TPM4*	Muscle contraction
			*BORCS8*	Heart development
			*CALR3*	Spermatogenesis
27	17.56–17.75	1.754087	*SGCZ*	Heart contraction
			*DLC1*	Brain development

**Fig. 3:**
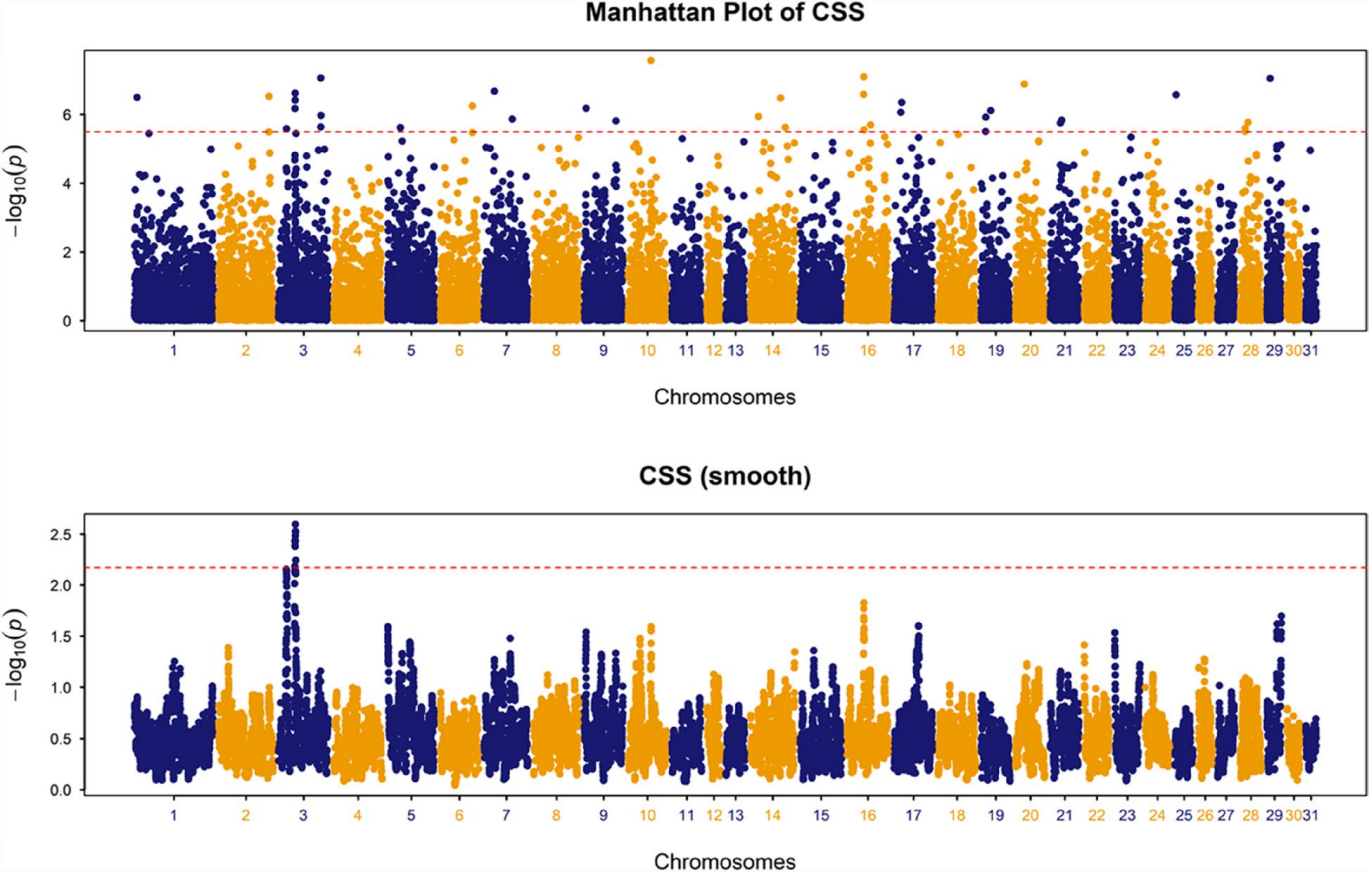
A chromosome-wide plot of composite selection signals (CSS) scores for the Asian (*n* = 133) vs. European horse (*n* = 191) populations in the dataset cohort. 1) Unsmoothed results, 2) Smoothed results, obtained by averaging the CSS scores of SNPs within 1 Mb sliding windows. The dashed red lines indicate the genome-wide 0.1% thresholds of the empirical scores. A single region with clusters of significant SNPs among the top 0.1% SNPs was identified on ECA3 containing the *ZFPM1* gene within the selection peak

On the other hand, comparison of the Asian versus European population groups localised a single significant genomic region on ECA3 that spanned ~ 1.5 Mb. The selection and its flanking regions contained 52 genes including genes that are related to lactation and grooming behaviour, mitochondria, inflammatory response, innate immune system, motor coordination, metabolic pathways, nervous system development, heart development, pigmentation, sperm mobility and gonad development, and intellectual disability (Fig. 3, Table 3, Table S7–8).

**Table 3:** Genes with functional significance identified in the selection peaks and flanking regions in the Asian versus European horse cohorts

ECA	Region (Mb)	CSS value	Candidate genes	Function
3	34.7–36.22	2.59	*APRT*	Lactation, grooming behaviour
			*CA5A*, *CTU2*, *MAP1LC3B*, *SPG7*, *ACSF3*, *CYBA*	Mitochondria
			*CYBA*, *IL17C*, *APRT*	Inflammatory response
			*GALNS*, *IL17C*	Innate immune system
			*JPH3*, *KLHDC4*, *SNAI3*	Motor coordination
			*MVD*	Metabolic pathways
			*SLC7A5*, *SPG7*	Nervous system development
			*ZFPM1*, *TCF25*, *FOXL1*, *FOXF1*, *FOXC2*, *DBNDD1*	Heart development
			*MC1R*	Pigmentation
			*GAS8*, *FANCA*	Sperm motility, gonad development
			*CDH15*, *ANKRD11*	Intellectual disability

Most strikingly, the selection peak contained the *ZFPM1* gene (Fig. S4), which overlaps with one of the two selection peaks identified among the domestic horse clade, DOM2, representing the earliest domesticates that became geographically widespread following 4200 YBP [17].

## Discussion

Tests for genome-wide selection signatures were performed using the CSS approach in regional subgroups of Chinese Mongolian and Asian horse breeds that have experienced long-term natural and artificial selection as discrete population units. This contrasts with previous studies that have identified selection signals for traits in domestic horse breeds that have experienced intense selection pressure applied over a relatively recent evolutionary time period [7, 8].

The major genetic determinants of the ability to perform an alternative form of gait (i.e. pace) and various coat colour traits in the horse are well understood and therefore provide phenotypes to demonstrate the utility of the CSS test in these populations. Here, the detection of a known genomic selection signal for gaitedness in the Wushen population suggests that this method can be used to identify other genomic regions containing genes for traits under selection within the studied populations.

The genomic region containing the *ASIP* gene on ECA22 was not immediately evident as a selection signature in Abaga Black, which has been bred for black coat colour (Fig. S1 and Table S2). However, when we included the top 1% SNPs, evidence for variation at the ECA22 locus containing *ASIP* was identified (Fig. S2, Table S3). The weaker than expected signal may be explained by the breeding history of this population. Although it is recorded that local herdsmen have favoured black horses since the thirteenth century and it is common to see black horses in this geographic area, the Abaga Black has only been officially recognised as a distinct breed since 2006, therefore the selection history may not be long enough to be captured by the initial stringent threshold criteria. Also, it is possible that there is a novel gene in this population contributing to the coat colour phenotype observed in this population that may be different from the common black coat colour observed in modern horse breeds [3]. Indeed, a number of rare coat colours have been identified in Mongolian horse populations that are not found in the more common modern European/American breeds [18]. Since several genomic regions on other chromosomes were identified in the Abaga Black, these genomic regions may harbour genes and/or genomic regulatory elements influencing the coat colour phenotype and other uncharacterised traits in this population.

### Selection signals for ‘Iron Hoof’

The equine hoof is a highly complex structure of the integument, which is produced by a modified epidermal layer covering the tip of the distal phalanx. The heritabilities of hoof shape and hoof wall quality phenotypes have previously been estimated as 0.12 and 0.10, respectively [9] indicating a modest genetic contribution to variation in hoof characteristics in the horse. Furthermore, a recent study that investigated the environmental effect on the morphometrics of the hooves of 100 feral Australian horses from five populations has suggested that a hard ground surface environment has an effect on the hooves, such as thickening of the hoof wall [10].

For the detection of selection signals in ‘Iron Hoof’, the *CSPG4* gene on ECA1 has been observed to be expressed in connective tissues, basement membranes and developing blood vessels [19]. Both *CSPG4* and *PEAK1* are expressed in equine hoof lamellar tissue [20] and involved in MMP-2 activity [21, 22], a member of the matrix metalloproteinases (MMP) family that is present in normal hoof wall lamellae [23]. The lamellar basement membrane changes may be the first step in lamellar failure occurring prior to detection with conventional methods [24]. Considering their function in MMP activity and expression in equine lamellar tissue, we hypothesise that genetic variation in *CSPG4* and *PEAK1* may influence MMP activity in the epidermal lamellae for maintenance of lamellar health. *CSPG4* could represent a strong candidate as it is also involved in dermal thickness, skin vascularization, vessel development [25] and keratinization [26].

There are several other candidate genes contained within the selection signals with functions that may be relevant to hoof biology. The selected region on ECA7 flanked the *EXPH5* gene, which is related to inherited skin fragility [27]. Six candidate genes on ECA3, *WWP2*, *PSMD7*, *NQO1*, *NOB1*, *NFAT5* and *HAS3*, are expressed in equine lamellar tissue and are likely to be involved in the biology of the hoof (Table 1). For example, in humans, a SNP (rs62051384) located within *WWP2* is associated with plantar fibromatosis, a rare fibrous hyperproliferation of the deep connective tissue of the foot [28]. A polymorphism (rs17336700) in the *PSMD7* gene is associated with ankylosing spondylitis in Chinese subjects [29]. The *NQO1* is a representative target of the nuclear factor, erythroid 2 like 2 (NFE2L2) transcription factor, which causes suppression of the macrophage inflammatory response [30]; inflammatory damage to the lamellae is one of the biological processes known to cause laminitis [31]. The protein product of *NFAT5* regulates synovial proliferation and angiogenesis in chronic arthritis [32]; the *NOB1*-encoded protein is likely to be a potential target for the treatment of osteosarcoma [33]; and the *HAS3* product is related to hyaluronan metabolism in human keratinocytes and atopic dermatitis skin [34].

Finally, several genes (*SEMA7A* [10], *CSK* [11], *GUCY1A2* [12], *EMCN* [13], *OPRM1* [14]) are associated with rheumatoid arthritis (RA). RA is a complex genetic disease that is relatively common and affects the joints with occasional skin manifestations [35]. However, it is unclear how this may relate directly to hoof strength, therefore further investigation of the association between RA-associated candidate genes and hoof strength may provide new insights to understand equine hoof physiology and possibly shed further light on RA in humans. Also, it may be possible that the Iron Hoof horses have been selected for traits other than the principle hard hooves phenotype.

### Selection signals relating to geographic origin

No genomic regions were common across the Asian versus European and Asian versus American/Middle Eastern population group comparisons. Only one selection signal was identified for the Asian versus American/Middle Eastern comparison. This selection signal contained the *HMGA2* gene, which has been reported to be associated with height variation and metabolic traits in ponies [36–38]. Generally, Asian horses tend to have smaller stature than western breeds. However, in the absence of additional phenotypic information for the horses used in this study, it is not possible to understand which trait the selection signal may be underpinning.

For the Asian versus European comparison, the selection peak for the highest ranked selection signal on ECA3 (35,533,253 bp) was closest to two genes, *ZNF469* (3:35654813–35,666,467 bp) and *ZFPM1* (35,728,941-35,748,261 bp) (Fig. S4). To our knowledge, there are no known associations for *ZNF469* among horse breeds. However, it is noteworthy that this selection signal overlaps with one of the two selection peaks identified among the domestic horse clade, DOM2. This clade represents the earliest domesticates that became geographically widespread following 4200 YBP [17]. The previously described selection peak at *ZFPM1* distinguished DOM2 from non-DOM2 archaeological specimens representing an early domestic group that did not contribute to modern horse populations. This selection signature has been hypothesised to reflect the emergence of docility and stress traits, which may reflect cognitive changes accompanying horse husbandry practices among intensively managed populations.

Two possible explanations may be proposed for the concordance of the *ZFPM1* selection peaks. First, extant Asian and European populations differ considerably in their mode of husbandry, with Mongolian horses being free-ranging, mimicking natural herd structures with little human interaction, and therefore they may have quite different cognitive requirements [1]. However, examination of genotype frequencies of the top SNP (chr3.35533253, rs68458737) among 806 horses from a range of global breeds (Table S9 and Fig. S5), suggests that the selection signal may be driven by native British Isles horse breeds rather than a behavioural phenotype distinctive of the Asian horses. The highest frequency of the GG genotype was observed in Shire (0.82), Clydesdale (0.71), Fell Pony (0.48) and Exmoor (0.33) and was absent or at low frequency (< 0.05) in six of the seven Asian breeds (Baicha Iron Hoof, Wuzhumuqin, Sanhe, Mongolian, Tuva and Wushen). Notably, Clydesdale and Shire are among the largest horse breeds [39], and the *ZC3H18* gene, located within the selection peak, is associated with body weight in other species [40]*.* However, an association of the G allele with body mass traits is not supported by the observation that Exmoor and Fell Pony breeds are small breeds and because the alternative homozygous genotype (AA) is fixed in the Belgian Draught sample.

We recently suggested that Mongolian horse populations may preserve ancient genomic variants that do not exist in other global horse breeds [5] as their paternal lineages can be dated to the early domesticates, at least 1400 YBP [41]. Therefore, a second possible explanation is that DOM2 horses did not replace all locally domesticated horse populations and Chinese Mongolian (and Mongolian and Tuva) horses may be the result of an entirely separate domestication event not linked to the Volga-Don origins. However, this is not supported by modelling of ancestral population structure in the Chinese Mongolian horses [5]. Consequently, based on these observations, a distinct contribution from native British Isles horses to modern horse genetic variation becomes plausible. Indeed, there is historical, morphological, and genetic evidence that has led to the assertion that wild horses survived in Great Britain and the Exmoor pony, for example, may be an ancient relic of pre-domestic horses [42]. Therefore, accurate reconstruction of the history of horse domestication using paleogenomes requires integration of sufficient genome-wide data from geographically representative modern horse populations, including older landrace or heritage breeds.

To further study regional adaptations in Chinese Mongolian horse populations, future research should focus on genome sequencing data for appropriate regional populations, rather than genotype data arising from SNP genotyping arrays. There can be considerable ascertainment bias in equine genomic studies that focus on the more common European and European-derived breeds. This is because the EquineSNP50 Genotyping BeadChip was designed predominately using information from polymorphic SNPs observed in seven European breeds [43], which has resulted in a genotyping array with low representation of rare variants within non-European breeds such as Asian horses. This ascertainment bias may therefore be responsible for the lower number of selection signatures detected using the 36 K SNP data set compared to the 511 K SNP data set. In addition, one of the component tests of CSS, the XP-EHH test, is haplotype-based and likely compromised by lower SNP densities [44].

## Conclusion

We have identified several genomic regions that underlie adaptive traits that have been shaped by long term microevolutionary processes in Asian horse populations. Importantly, plausible candidate genes that may be involved in hoof health have been identified in the Baicha Iron Hoof horse. The Baicha Iron Hoof is a critically endangered breed and the identification of genetic variants under selection in this population, that distinguish them from other local populations, emphasises the need for efforts to protect and conserve ancient, heritage and landrace livestock with distinct microevolutionary histories. In addition, the signal of selection detected in Asian populations highlights the importance of inclusion of modern breeds in the interpretation of paleogenomic data for inference of population history. The coincidence of a selection peak among modern horse breeds at an evolutionarily critical locus, raises the question whether DOM2 horses replaced all locally domesticated horses, or whether some other archaic lineages still survive, with their ancestors not yet identified.

## Methods

### DNA samples and genotypic data

Methods for sample collection were performed in accordance with approval from the University College Dublin Animal Research Ethics Committee (AREC-E-15-14-Hill). Tail hair samples were collected from horses and 155 horses from five distinct Chinese Mongolian populations were genotyped using the Axiom™ MNEC670 Equine Genotyping Array (Applied Biosystems/ThermoFisher Scientific). Following removal of closely related horses (pi_hat > 0.3 in PLINK v1.9) [45], 100 horses were included for analysis. Sample information and SNP data are described in detail in [5]. After SNP quality control (genotype missing rate > 0.05 and MAF < 0.01) 511,913 autosomal SNPs that mapped to EquCab 3.0 were used for the Chinese Mongolian population genomics analyses.

To expand the number of comparator horse populations, publicly available genotypes generated from the Illumina EquineSNP50 Genotyping BeadChip were obtained and merged with the Chinese Mongolian horse data. After SNP quality control (genotype missing rate > 0.05 and MAF < 0.01), 36,351 autosomal SNPs that mapped to EquCab 3.0 were retained. The publicly available horse samples and SNP genotypes have been previously reported [46] and the data were obtained from www.animalgenome.org/repository/pub/UMN2013.0125. Imputation of missing genotypes and haplotype phasing were performed using BEAGLE 3.3 [47].

### Composite selection signature (CSS) cohorts of contrasting phenotypes of interest

The phenotypes for the five Chinese Mongolian horse populations were documented and photographs of each horse taken at the time of sample collection. The detailed information has been previously described [5]. Contrasting cohorts selected from additional global breeds were defined to identify signatures of selection. The overall comparison cohorts are shown in Table S10.

To validate the CSS approach, two datasets (A and B) were created from the Chinese Mongolian populations (511 K SNPs) to evaluate selection for traits known to be under the control of single genes with major effects, namely gait and black coat colour. Dataset A included a single gaited horse population (Wushen) and four non-gaited horse populations. A SNP (23:22359351) tagging the *DMRT3* gene located on ECA23 (ECA23: 22,378,399 – 22,392,510) is highly associated with the ability to pace in horses [9]. Dataset B consisted of animals from a population selected for black coat colour (Abaga Black) and four non-black coat colour populations. The *ASIP* gene for black coat colour is located on ECA22 (ECA22: 26,009,341 – 26,072,655). Horses that are homozygous for an 11 bp deletion in exon 2 of the *ASIP* gene are black in colour [48].

Dataset C (511 K SNPs) included the ‘Iron Hoof’ population (Baicha Iron Hoof) and four non-Iron hoof horse populations; Dataset D and dataset E (36 K SNPs) were generated to identify signatures of selection in Asian populations (five Chinese Mongolian horse populations and two publicly available Asian populations) compared with American/Iberian/Middle Eastern and European populations: dataset D was for Asian versus American/Iberian/Middle Eastern; dataset E was for Asian versus European.

### Composite selection signal (CSS) method

The composite selection signals (CSS) approach was developed to investigate genomic signatures of selection and has been successful at localising genes for monogenic and polygenic traits under selection in livestock [8, 49]. As the CSS uses fractional ranks of constituent tests and does not incorporate the statistics with *P-*values, it allows a combination of the evidence of historical selection from different selection tests. For the present study, the CSS combined the fixation index (*F*_ST_), the change in selected allele frequency (Δ*SAF*) and the cross-population extended haplotype homozygosity (*XP-EHH*) tests into one composite statistic for each SNP. *F*_ST_ statistics were computed as the differentiation index between the population/s of interest (as selected) and the contrasting/reference population/s (as non-selected). *XP-EHH* and Δ*SAF* statistics were computed for the selected population/s against the reference population (as non-selected). The composite selection statistics (CSS) were computed as followed by [8]. To reduce spurious signals, the individual test statistics were averaged (smoothed) over SNPs across chromosomes within 100 kb sliding windows for the 511 K SNP data and 1 Mb for the 36 K SNP data, respectively.

### Identification of selected genomic regions, candidate gene mining

To localise genomic regions and genes under selection, for the both SNP data sets (~ 511 K SNPs and ~ 36 K SNPs), significant genomic regions (called as cluster regions) were defined as those that harbour at least one significant SNP (top 0.1%) surrounded by at least five SNPs among the top 1%. SNPs among the top 0.1% smoothed CSS values within the sliding windows were considered significant. Consecutive clusters spaced < 1 Mb apart were merged into a single cluster. Genes underlying the selection peaks as well as flanking regions (±0.5 Mb) were mapping to an annotated protein-coding gene list from EquCab3.0 downloaded from Ensembl. These genes were then examined for function annotation using review of literature, DAVID functional annotation tool [50, 51] and the GeneCards database [52].

## Supplementary Information

**Additional file 1: Figure S1.** A chromosome-wide plot of composite selection signals (CSS) scores for Abaga Black vs. other Chinese Mongolian populations (top 0.1% SNPs threshold). **Figure S2.** A chromosome-wide plot of composite selection signals (CSS) scores for Abaga Black vs. other Chinese Mongolian populations (top 1% SNPs threshold). **Figure S3.** A chromosome-wide plot of composite selection signals (CSS) scores for Asian vs. American/Middle Eastern populations. **Figure S4.** Composite selection signals (CSS) scores for individual SNPs at the ECA3 selection peak (Asian vs. European populations) showing peak close to the ZFPM1 gene. **Figure S5.** Genotype frequencies of top SNP on ECA3 (chr3.35533253, rs68458737) in a range of horse breeds. The highest frequency of the GG genotype was among British Isles horse breeds.

**Additional file 2: Table S1.** Composite Selection Signals (CSS) results for Wushen vs. other Chinese Mongolian populations. **Table S2.** Composite Selection Signals (CSS) results for Abaga Black vs. other Chinese Mongolian populations (top 0.1% SNPs threshold). **Table S3.** Composite Selection Signals (CSS) results for Abaga Black vs. other Chinese Mongolian populations (top 1% SNPs threshold). **Table S4.** Composite Selection Signals (CSS) results for Iron Hoof vs. other Chinese Mongolian populations (top 0.1% SNPs threshold). **Table S5.** Composite Selection Signals (CSS) results for Asia vs. American/ Middle Eastern horse. **Table S6.** Functional annotation table for the genes identified within the selection signals in the comparison Asia vs. American/Middle Eastern horse. **Table S7.** Composite Selection Signals (CSS) results for Asian vs. European horse. **Table S8.** Functional annotation table for the genes identified within the selection signals in the comparison Asian versus European horse. **Table S9.** Genotype frequencies for the top SNP (chr3.35533253, rs68458737) were calculated for a range of global horse breeds. **Table S10.** Traits, contrasting groups, number of populations and individuals, genotype information (SNPs), known genes for the traits of interest and dataset code.

## Data Availability

The data generated in this study has been deposited in the European Variation Archive (Project ID: PRJEB55177; the link to access: https://www.ebi.ac.uk/eva/?eva-study=PRJEB55177).

## Abbreviations

IMAR: Inner Mongolia Autonomous Region
XUAR: Xinjiang Uygur Autonomous Region
SNP: Single-nucleotide polymorphism
PCA: Principal component analysis
PCs: Principal components
*F*_ST_: Pairwise fixation index
CSS: Composite selection signals
MMP: The matrix metalloproteinases
Δ*SAF*: Selected allele frequency
*XP-EHH*: Extended haplotype homozygosity
