# Supplementary material for: Selection signatures for local and regional adaptation in Chinese Mongolian horse breeds reveal candidate genes for hoof health
Source: BMC Genomics. 2023 Jan 19;24:35. doi: 10.1186/s12864-023-09116-8 (PMC9854188; doi:10.1186/s12864-023-09116-8)
Supplement: Supplementary file 1 — Additional file 1: Figure S1. A chromosome-wide plot of composite selection signals (CSS) scores for Abaga Black vs. other Chinese Mongolian populations (top 0.1% SNPs threshold). Figure S2. A chromosome-wide plot of composite selection signals (CSS) scores for Abaga Black vs. other Chinese Mongolian populations (top 1% SNPs threshold). Figure S3. A chromosome-wide plot of composite selection signals (CSS) scores for Asian vs. American/Middle Eastern populations. Figure S4. Composite selection signals (CSS) scores for individual SNPs at the ECA3 selection peak (Asian vs. European populations) showing peak close to the ZFPM1 gene. Figure S5. Genotype frequencies of top SNP on ECA3 (chr3.35533253, rs68458737) in a range of horse breeds. The highest frequency of the GG genotype was among British Isles horse breeds. [file 12864_2023_9116_MOESM1_ESM.docx]

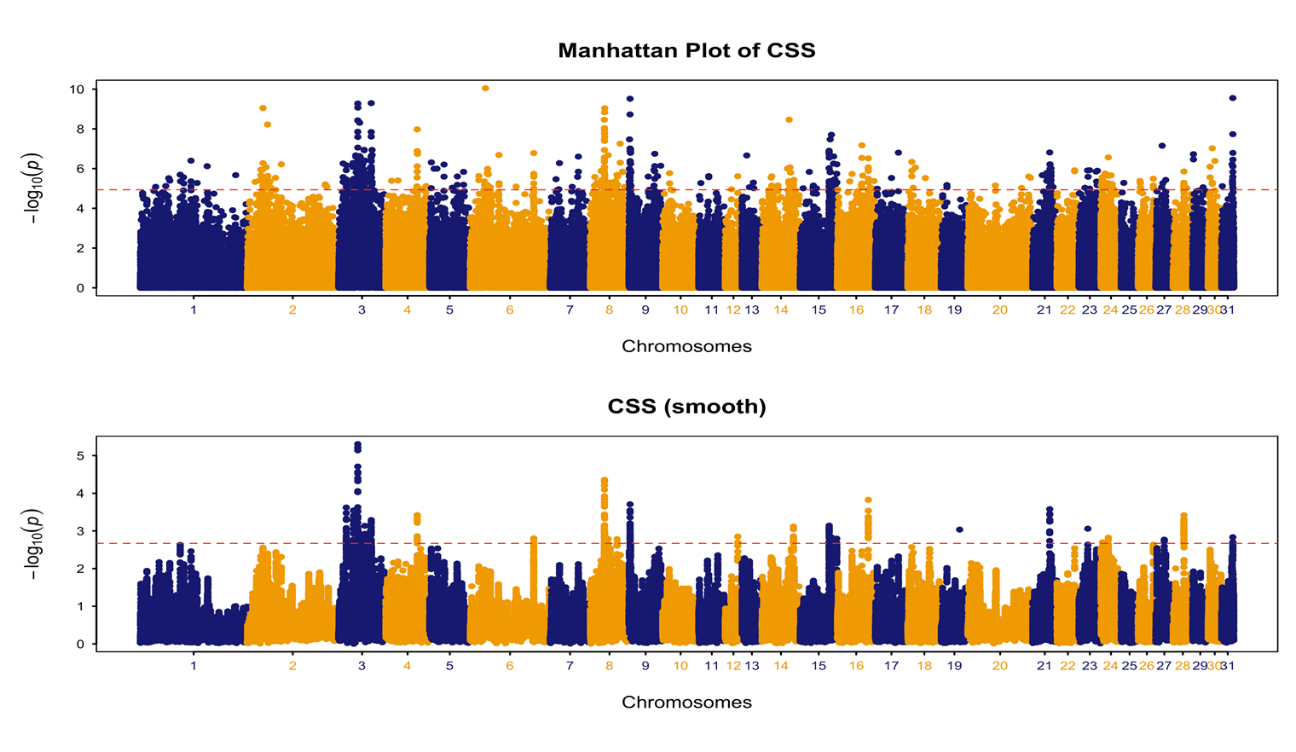


**Figure S1.** A chromosome-wide plot of composite selection signals (CSS) scores for Abaga Black vs. other Chinese Mongolian populations (top 0.1% SNPs threshold)


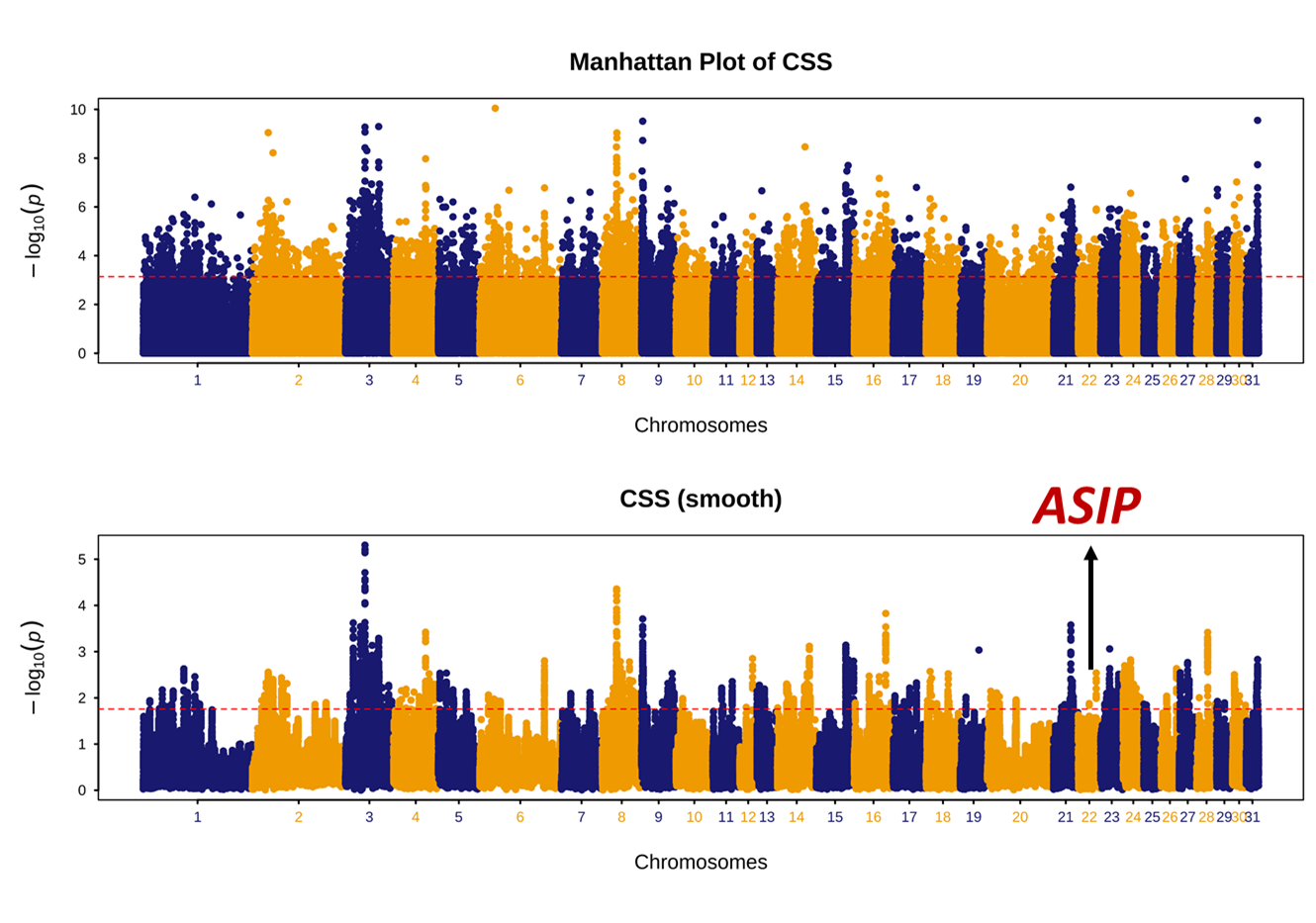


**Figure S2.** A chromosome-wide plot of composite selection signals (CSS) scores for Abaga Black vs. other Chinese Mongolian populations (top 1% SNPs threshold)


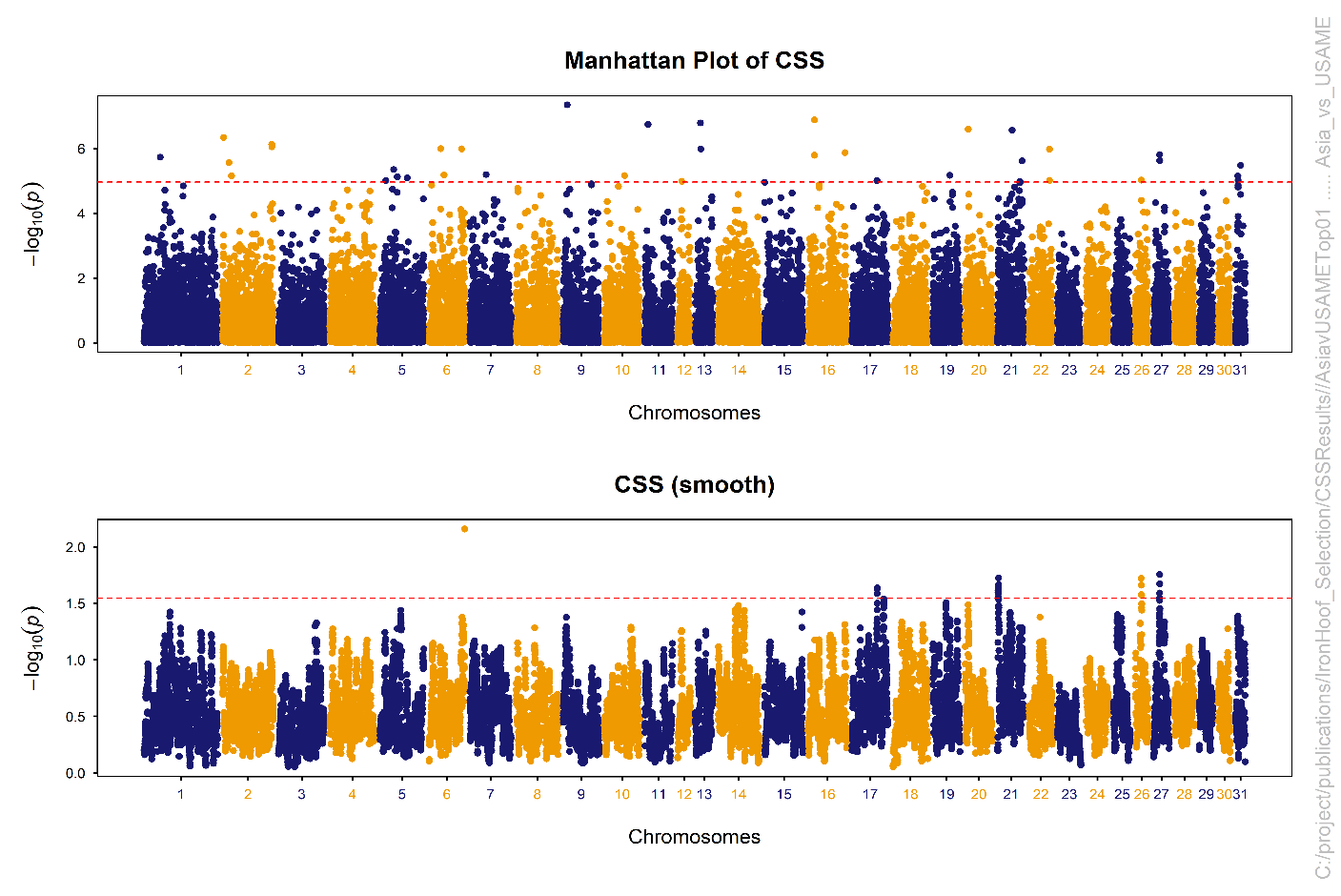


**Figure S3.** A chromosome-wide plot of composite selection signals (CSS) scores for Asian vs. American/Middle Eastern populations


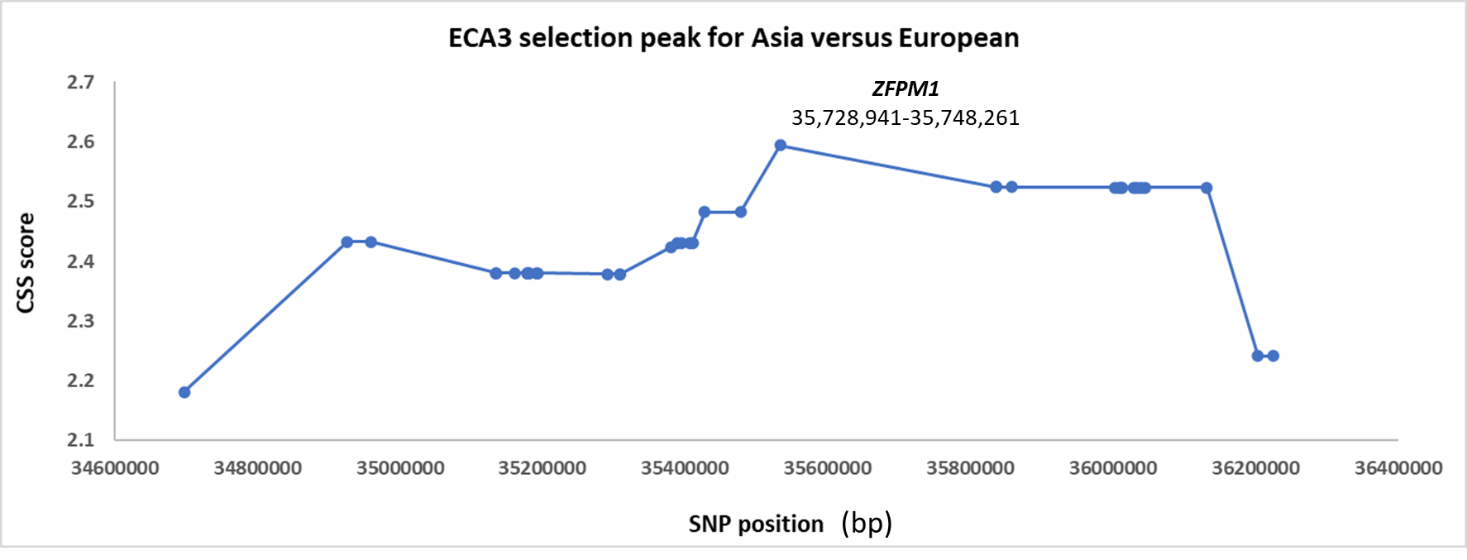


**Figure S4.** Composite selection signals (CSS) scores for individual SNPs at the ECA3 selection peak (Asian vs. European populations) showing peak close to the ZFPM1 gene.


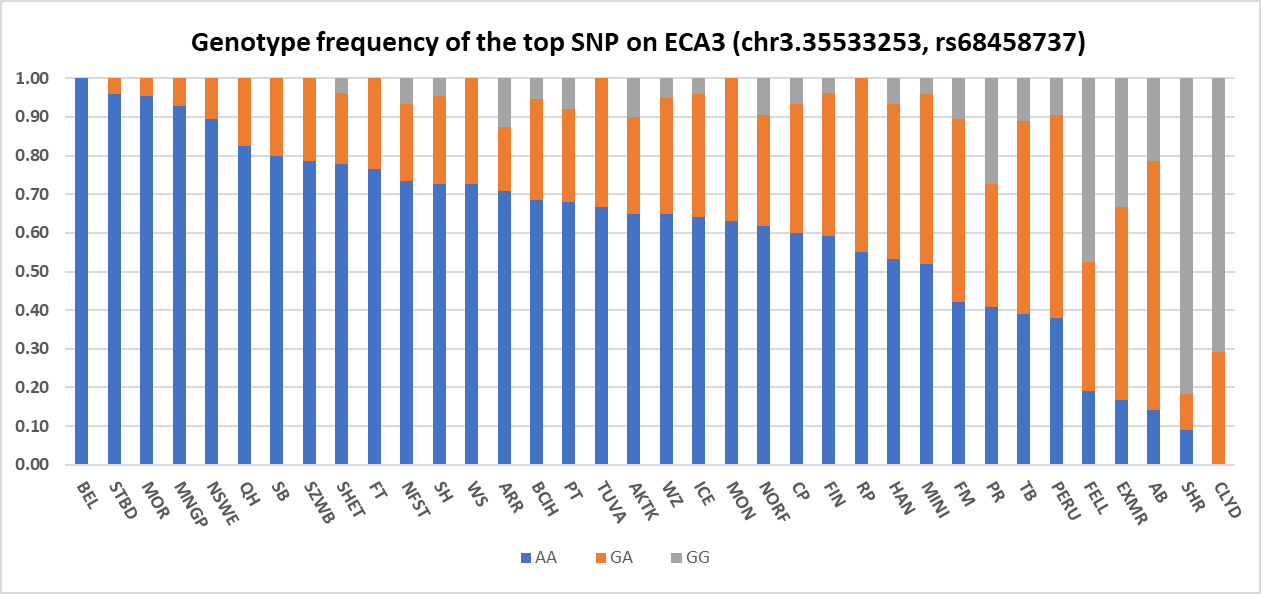


**Figure S5.** Genotype frequencies of top SNP on ECA3 (chr3.35533253, rs68458737) in a range of horse breeds. The highest frequency of the GG genotype was among British Isles horse breeds.
